# Osteoporotic fracture rates in chronic hemodialysis and effect of heparin exposure: a retrospective cohort study

**DOI:** 10.1186/s12882-020-01916-4

**Published:** 2020-07-09

**Authors:** Hind Harrak, Emilie René, Noor Alsalemi, Naoual Elftouh, Jean-Philippe Lafrance

**Affiliations:** 1 Centre de recherche Hôpital Maisonneuve-Rosemont, Montréal, Canada; 2 Service de néphrologie, Hôpital Maisonneuve-Rosemont, 5415, boul. de l’Assomption, Montréal, Québec H1T 2M4 Canada; 3 Département de pharmacologie et physiologie, Université de Montréal, Montréal, Canada

**Keywords:** Heparin, low molecular weight, Unfractionated heparin, Osteoporotic fracture, Kidney failure, chronic, Pharmacoepidemiology

## Abstract

**Background:**

Patients receiving chronic hemodialysis treatments are at a higher risk of fracture compared to the general population. While the use of heparin during dialysis is crucial to avoid thrombosis of the extracorporeal circuit, the association of unfractionated heparin (UFH) and the risk of osteoporotic fracture has been shown for many years. However, this association was not as clear for low-molecular-weight heparin (LMWH) and the few collected data originated from studies among pregnant women. Our aim was to measure osteoporotic fracture rate among hemodialysis patients and to evaluate the association of LMWH compared to UFH in hemodialysis.

**Methods:**

A retrospective cohort study was conducted on data extracted from the RAMQ and Med-Echo databases from January 2007 to March 2013 with patients chronically hemodialyzed in 21 participating centers. Incidence rates for each fracture sites were measured per 1000 patient-year (p-y) and their 95% confidence intervals (CI). Osteoporotic fracture risk for a first event with LMWH compared to UFH was estimated using a cox proportional hazard model using demographics, comorbidities and drug use as covariates.

**Results:**

4796 patients undergoing chronic hemodialysis were identified. The incidence rate for all fracture sites was 22.7 /1000 p-y (95% CI: 19.6–26.1) and 12.8 /1000 p-y (95% CI: 10.5–15.4) for hip and femur fractures. We found a similar risk of osteoporotic fracture for LMWH compared to UFH (adjusted HR = 1.01; 95%CI: 0.72–1.42). Age and malignancy increased the risk of fracture while cerebrovascular disease decreased the risk of fracture.

**Conclusions:**

Compared to UFH, LMWH did not change the risk of osteoporotic fracture when used for the extracorporeal circuit anticoagulation in chronic hemodialysis.

## Background

Hospitalization and mortality following a bone fracture are significantly higher among hemodialysis patients compared to the overall population [1]. Not only hemodialysis patients have worse outcomes after a fracture, they are also at a higher risk of bone disease and fracture [2–6]. Bone structure and function in these patients is altered by mineral and endocrine disorders [7]. Heparin plays a central role in preventing thrombosis of the extracorporeal circuit in hemodialysis. However, it was reported in animal models and human studies that heparin can induce osteoporosis [8–17]. The exact mechanism is still under investigation, but few hypotheses have been expressed. One possible reason for heparin-induced osteoporosis would be that heparin decreases the bone density by decreasing the number of osteoblasts and increasing the number of osteoclasts [18]. Another explored option was the interaction between heparin and the system composed of osteoprotegerin (OPG), the receptor activator of nuclear factor kB (RANK), and the receptor activator of nuclear factor kB ligand (RANKL), which is essential in bone remodeling [19–25]. It is still unclear if the induction of osteoporosis varied between unfractionated heparin (UFH) and low-molecular-weight heparin (LMWH) and, furthermore, if the use of one form of heparin instead of the other changed the risk of fracture. Since the fracture risk is high among chronic hemodialysis patients and that they are usually chronically exposed to a therapeutic dose of heparin three times weekly during their hemodialysis treatment, a small relative reduction in the fracture risk may translate in the prevention of substantial number of fractures.

The aims of our study were 1) to evaluate fracture rates in a cohort of chronic hemodialysis patients; and 2) to measure the association between the type of heparin (LMWH compared to UFH) and the risk of osteoporotic fractures.

## Methods

### Study population and data sources

We used a retrospective cohort study to measure osteoporotic fracture rates and to evaluate the association of osteoporotic fracture risk and exposure to LMWH compared to UFH among prevalent and incident chronic hemodialysis patients. Data were obtained from the *Régie de l’assurance maladie du Québec* (RAMQ), a provincial health insurance plan of the Province of Québec, Canada, provided to all residents. This single-payer plan covers medical and hospital services. This administrative database provided all medical visits, diagnostic codes (using International Classification of Diseases – ICD), medical procedures during in- and outpatient encounters, and hospital discharge summaries (Med-Echo). The Med-Echo database holds details on hospital stay, including the date of admission and discharge, primary and secondary diagnoses, and the procedures performed. The provincial drug plan covers all individuals, including workers with no private insurance, aged 65 years and older, and individuals receiving welfare. RAMQ drug plan does not report exposure to heparin during hemodialysis and therefore this information was collected at each of the 21 participating hemodialysis units in the province of Quebec. The list of participating centers is provided in the supplementary appendix.

### Study cohort

Our cohort is formed of both prevalent and incident adult patients on maintenance hemodialysis between January 1st, 2007 and March 31st, 2013 identified in the RAMQ database. Inclusion criteria are defined as follow: patients could not have a prior kidney transplant and should have at least 90 days of follow-up after hemodialysis initiation. The cohort entry date corresponds to the first hemodialysis date respecting the inclusion criteria. However, follow-up began only when a patient was exposed to one form of heparin for at least 3 months after cohort entry (index date). End of follow-up was defined as the date of kidney transplant, switch to peritoneal dialysis or home dialysis, switched heparin form or with unknown heparin exposure status, end of study or death, whichever occurred first.

### Exposure definition

Anticoagulant administration is part of the hemodialysis session and is performed in hospital. Therefore, the type of heparin received by the patient is not available in the RAMQ drug plan database. Each patient was assigned to the heparin regimen that corresponded to the unit’s common protocol. We collected the type of heparin used (tinzaparin, dalteparin, enoxaparin, nadroparin, or UFH) for each center between January 1st, 2007 and March 31st, 2013. Protocol changes were documented allowing for switch between heparin regimen through the study period. Multiple changes of heparin could be reported for each hemodialysis center, however transition periods from one heparin to another were removed from the analysis. Patients’ exposure status was assigned based on the center where they were receiving hemodialysis.

### Outcome definition

We used a validated algorithm published by Jean et al. [26] to identify incident osteoporotic fracture events during patients’ follow-up time, using a combination of physician claims and ICD-10 codes. Briefly, the algorithm identifies incident fractures through two ways: 1) a physician claim that is specific to fracture treatment: closed reduction, open reduction or immobilization; 2) a billing code for a medical visit with an osteoporotic surgeon combined with some specific ICD-10 diagnostic codes and another claim for a principal visit (emergency physician or general practitioner) with some specific ICD-10 diagnostic codes. The list of billing claim codes and ICD-9/ICD-10 codes provided in supplementary Tables S1 and S2. A total of 12 osteoporotic fracture sites and one unspecified osteoporotic fracture site categories were evaluated in our cohort. Only the first fracture was considered for each patient.

### Covariates

The following covariates were evaluated at the index date: age, gender, follow-up time, vintage time (time undergoing chronic hemodialysis for prevalent patients), hospitalization in prior year, comorbidities in the 2 years prior to index date, and drug use in the 6 months prior to the index date (see Table 1 for more details).

### Statistical analysis

Baseline data were described as mean and standard deviation (SD) or median and interquartile range (IQR) a where appropriate. Comorbidities are presented as frequency and proportions (%).

Incidence rates for outcomes were calculated by dividing the number of events (total osteoporotic fractures or osteoporotic fractures by site) by the total patient-years (p-y) of follow-up. 95% confidence intervals (CI) for rates were calculated using a Poisson distribution (inversed gamma formula).

The hazard ratio (HR) for the first fracture per patient was estimated using a cox proportional hazard model. It was adjusted for all the comorbidities presented in Table 1. All analyses were done using SAS 9.4 (Cary, North Carolina).

### Sensitivity analysis

LMWH differ from one another and their effect on bone metabolism could vary. We conducted the analyses using the same method but separating tinzaparin periods from dalteparin (UFH was kept as the reference group). Finally, we performed an additional analysis including either only incident patients or prevalent patients from our cohort.

### Ethical considerations

This study was approved by the Government of Québec ethics committee (*Commission d’accès à l’information*) and all hospitals ethics committees. Informed consent was waived.

## Results

A total of 4796 incident and prevalent patients on maintenance hemodialysis were identified between January 1st, 2007 and March 31st, 2013. Median follow-up time was 1.95 years (IQR: 0.87–3.68) for the total cohort, with incident patients representing 68.9% of the patients. The mean age after 3 months of exposure was 67.0 ± 14.0 years and women represented 39.7%. LMWH patients represented 30% of the cohort. When comparing the proportion of patients receiving LMWH and UFH by year of cohort entry, 22% of patients were receiving LMWH between 2007 and 2009. In 2010, 35% of the patients were receiving LMWH and 50% of patients entering in the cohort in 2011–2012 were receiving LMWH. Patients’ characteristics were overall similar (Table 1). However, patients in the LMWH group were older, were more incident than prevalent, had more hypertension, less history of parathyroidectomy and slightly different prescription drugs.

**Table 1:** Characteristics of patients receiving LMWH compared to UFH in hemodialysis

Covariates	LMWH	UFH	*p* value
	*n* = 1426 (%)	*n* = 3370 (%)	
Age^a^ (years) ± SD	67.88 ± 13.7	66.57 ± 14.2	0.003
Sex (Female)	589 (41.3)	1316 (39.1)	0.14
Hemodialysis incidence	1090 (76.4)	2215 (65.7)	< 0.0001
Hospitalization in prior year	1117 (78.3)	2678 (79.5)	0.38
Comorbidities^b^			
Cardiovascular disease	757 (53.1)	1836 (54.5)	0.38
Cerebrovascular disease	121 (8.5)	315 (9.4)	0.34
Chronic pulmonary disease	324 (22.7)	711 (21.1)	0.21
Chronic liver disease	72 (5.1)	213 (6.3)	0.09
Congestive heart failure	473 (33.2)	1051 (31.2)	0.18
Diabetes	796 (55.8)	1823 (54.1)	0.27
Hyperlipidemia	876 (61.4)	2132 (63.3)	0.23
Hypertension	1169 (82.0)	2614 (77.6)	0.001
Malignancy	289 (20.3)	679 (20.2)	0.93
Peripheral vascular disease	407 (28.5)	992 (29.4)	0.53
Rheumatoid arthritis	22 (1.5)	66 (2.0)	0.33
Osteoporosis	92 (6.5)	205 (6.1)	0.63
Parathyroidectomy	0 (0.0)	10 (0.3)	0.04
Prior fracture	34 (2.4)	100 (3.0)	0.26
Drug use^c^			
NSAIDs	743 (52.1)	1763 (52.3)	0.89
Steroids	212 (14.9)	565 (16.8)	0.10
Calcium	872 (61.2)	2121 (62.9)	0.24
Vitamin D	801 (56.2)	1750 (51.9)	0.01
Phosphorus chelating agents	914 (64.1)	2249 (66.7)	0.08
Cinacalcet	14 (1.0)	18 (0.5)	0.08

^a^At index date

^b^At index date in the two years prior

^c^At index date in the six months prior

Abbreviations: *SD* Standard deviation, *NSAID* Non-steroidal anti-inflammation drugs, *LMWH* Low Molecular Weight heparin, *UFH* Unfractionated heparin

The incidence rate for a first fracture, when all sites were combined, was 22.7 /1000 p-y (95% CI: 19.6–26.1) with hip and femur fractures being the most common with a rate of 12.8 /1000 p-y (95% CI: 10.5–15.4). Rates of first fracture for all included sites are provided in Table 2. Fracture rates were similar for both LMWH and UFH groups.

**Table 2:** Incidence rates for the first fracture by site (per 1000 person-year)

Site	All	LMWH	UFH	*p* value
	Rate (95%CI)	Rate (95%CI)	Rate (95%CI)	
All Sites	22.7 (19.6–26.1)	22.5 (16.5–29.9)	22.7 (19.2–26.7)	0.95
Ankle	1.5 (0.8–2.6)	1.4 (0.3–4.1)	1.5 (0.7–2.8)	0.89
Hip and Femur	12.8 (10.5–15.4)	14.7 (10.0–20.8)	12.1 (9.6–15.1)	0.39
Foot	0.5 (0.1–1.2)	*n/a*	0.6 (0.2–1.6)	
Forearm fracture	1.5 (0.8–2.6)	1.4 (0.3–4.1)	1.5 (0.7–2.8)	0.8864
Humerus	0.8 (0.3–1.7)	1.4 (0.3–4.1)	0.6 (0.2–1.6)	0.3630
Knee	0.7 (0.3–1.5)	0.9 (0.1–3.4)	0.6 (0.2–1.6)	0.6603
Pelvis	0.8 (0.3–1.7)	0.5 (0.0–2.6)	0.9 (0.3–2.0)	0.4486
Shoulder fracture	0.7 (0.3–1.5)	0.5 (0.0–2.6)	0.8 (0.2–1.8)	0.6033
Spine	0.8 (0.3–1.7)	*n/a*	1.1 (0.4–2.2)	
Tibia	0.5 (0.1–1.2)	*n/a*	0.6 (0.2–1.6)	
Upper limb	*n/a*	*n/a*	*n/a*	
Wrist	1.3 (0.6–2.3)	1.4 (0.3–4.1)	1.2 (0.5–2.4)	0.85
Unspecified	0.5 (0.1–1.2)	*n/a*	0.6 (0.2–1.6)	

Abbreviations: *LMWH* Low Molecular Weight heparin, *UFH* Unfractionated heparin, *CI* Confidence interval

Using a multivariable model, the fracture risk was also similar for LMWH compared to UFH (HR = 1.01; 95%CI: 0.72–1.42). However, older age (HR = 1.02; 95% CI: 1.01–1.04) and malignancy (HR = 1.50; 95% CI: 1.07–2.10) were associated with a higher risk of fracture. Cerebrovascular disease was associated with a lower risk of fracture (HR = 0.46; 95% CI: 0.24–0.89). Detailed results are presented in Table 3.

**Table 3:** Estimated risk of osteoporotic fracture associated with LMWH in hemodialysis

Variables	Unadjusted HR (95% CI)	Adjusted HR (95% CI)
Baseline		
Heparin Exposure LMWH (vs UFH)	1.00 (0.72–1.39)	1.01 (0.72–1.42)
Age (per year)	1.02 (1.01–1.04)	1.02 (1.01–1.04)**
Male (vs female)	1.24 (0.94–1.65)	1.21 (0.90–1.62)
Incident patients (vs prevalent)	0.89 (0.67–1.20)	0.84 (0.61–1.16)
Hospitalization in prior year	1.11 (0.79–1.55)	1.14 (0.77–1.70)
Comorbidities		
Cardiovascular disease	1.14 (0.85–1.51)	0.91 (0.65–1.28)
Cerebrovascular disease	0.58 (0.30–1.09)	0.46 (0.24–0.89)*
Chronic pulmonary disease	1.30 (0.93–1.82)	1.20 (0.84–1.71)
Chronic liver disease	1.10 (0.60–2.02)	1.25 (0.67–2.31)
Congestive heart failure	1.34 (1.00–1.81)	1.24 (0.88–1.75)
Diabetes	1.20 (0.90–1.60)	1.25 (0.92–1.72)
Hyperlipidemia	1.22 (0.90–1.66)	1.16 (0.82–1.65)
Hypertension	0.96 (0.69–1.34)	0.75 (0.51–1.11)
Malignancy	1.53 (1.10–2.12)	1.50 (1.07–2.10)*
Peripheral vascular disease	1.29 (0.95–1.75)	1.29 (0.92–1.81)
Rheumatoid arthritis	1.73 (0.71–4.21)	1.51 (0.61–3.75)
Osteoporosis	1.48 (0.87–2.51)	1.20 (0.69–2.09)
Prior fracture	1.57 (0.77–3.18)	1.33 (0.64–2.76)
Drug use 6 months prior		
NSAID	1.03 (0.77–1.37)	0.87 (0.63–1.20)
Steroids	1.04 (0.70–1.54)	0.97 (0.64–1.46)
Calcium	1.04 (0.77–1.41)	1.07 (0.62–1.85)
Vitamin D	1.32 (0.99–1.77)	1.37 (0.98–1.92)
Phosphorus chelating agents	0.96 (0.70–1.30)	0.71 (0.41–1.26)
Cinacalcet	0.53 (0.07–3.80)	0.62 (0.09–4.47)

Abbreviations: *HR* Hazard ratio, *CI* Confidence interval, *NSAID* Non-steroidal anti-inflammation drugs, *LMWH* Low Molecular Weight heparin, *UFH* Unfractionated heparin

*: *p* value < 0.05; **: *p* value < 0.01

### Sensitivity analysis

When comparing single LMWH agent to UFH, neither tinzaparin (HR = 0.94; 95% CI: 0.66–1.34) nor dalteparin (HR = 2.35; 95% CI: 0.91–6.03) were associated with a different fracture risk than UFH. In the second sensitivity analysis where only incident patients were included, the fracture risk remained similar when comparing LMWH to UFH (HR = 0.89; 95% CI: 0.58–1.38). Our final sensitivity analysis including only prevalent patients showed no association between the type of heparin and fracture risk (HR = 1.07; 95% CI: 0.60–1.91).

## Discussion

In this retrospective cohort, we evaluated the incidence rate of osteoporotic fractures among chronic hemodialysis patients. We also estimated the risk of fracture associated with LMWH and differentiating between tinzaparin and dalteparin. Regardless of the type of LMWH administered, there was no difference in fracture risk compared to UFH.

Fracture rates from our cohort are consistent with rates presented by Jadoul et al. from the second phase of the Dialysis Outcomes and Practice Patterns Study (DOPPS II) which includes data from 12 countries [4]. The authors reported a total incidence rate for any fracture of 25.6 events /1000 p-y (95%CI: 24.4–27.0) and 8.9 events /1000 p-y (95% CI: 8.4–9.4) for hip fracture. Both our results and results from DOPPS II show higher fracture incidence rates compared to the general population that showed hip fracture rates varying between 1 and 5 events /1000 p-y [27].

Our results show no fracture risk difference with LMWH compared to UFH among chronic hemodialysis patients. Case reports and studies reporting an association between UFH and osteoporosis and osteoporotic fractures have been discussed for more than 50 years, [8] but most data are from pregnant women using long term heparin to prevent pregnancy loss [28, 29]. Even in this population, the association of LMWH and osteoporosis remains controversial [30, 31]. The proposed mechanism to explain the reduction of osteoporosis associated with LMWH is the following: While both LMWH and UFH decrease osteoblast count, UFH would trigger a higher increase of osteoclast surface and a greater loss of calcium than LMWH [18, 32].

In our cohort, age was associated with an increased risk of fracture. In a recent study by Wagner et al. [33] using data from the US Renal Data System (USRDS), reported that white patients aged 65 years or more undergoing hemodialysis had higher fracture rates compared to other age groups. We also observed an increased risk of fracture associated with malignancy. Multiple studies reported higher risk of fracture among patients with bone cancer, multiple myeloma, metastases to the bone and organs other than the bone, liver, gall bladder, pancreas, breast and other forms of cancer [34–38]. This increased risk could be explained by multiple factors like cancer treatments used [39–41], infiltration of cancer in bone tissue, a result of the systemic inflammation or parathyroid activity [42]. Our data showed a lower risk of fracture for patients with a history of cerebrovascular disease. However, previous studies found no association between cerebrovascular disease and risk of fracture, or reported an increased risk [43–46]. Why this result differs from previous studies remains unknown. A possible explanation could be that patients with cerebrovascular disease receive more intensive care with better control of fracture risk factors than patients without cerebrovascular disease [45]. Further studies on this topic are needed to clarify this association.

To our knowledge, this is the first study evaluating the risk of fracture associated with LMWH compared to UFH among chronic hemodialysis patients. Multiple studies reported on the risk of fracture in hemodialysis patients, or on heparin-induced osteoporosis, but none studied the association between type of heparin and the risk of fracture in the hemodialysis setting. Our study has several strengths. Outcomes were evaluated on large scale in this multicenter cohort study. The universal provincial health care insurance allowed including all eligible patients undergoing hemodialysis in participating units, limiting selection bias. Multiple covariates, including drug exposure, were collected from RAMQ and Med-echo and included in the analyses, and therefore minimizing confounding. Finally, we used a validated algorithm to identify osteoporotic fractures in the cohort. This algorithm was specifically designed for RAMQ data and showed an overall high sensitivity and positive predictive value.

Our study has some limitations. Exposure data was collected at the facility level and patients were attributed their exposure status based on the facility where they received hemodialysis treatment, introducing a potential misclassification bias. The proportion of patients who were not receiving the standard heparin regimen was not available. Since we excluded transition period, this proportion is thought to be small. Nevertheless, this bias should not be different between LMWH and UFH. Moreover, individual dosage was not available, limiting this adjustment in the analysis. Despite the validated algorithm to identify fracture events, some fractures may have been missed, especially for vertebral, sacral and coccyx fracture sites that had the lowest sensitivities. The algorithm was independent from the exposure status and should not influence the risk association. While we included numerous relevant covariables in our model, residual confounding is possible. If available, patients’ biochemical profiles would have been valuable in our current study. Finally, only a small portion of patients were exposed to dalteparin, limiting the interpretation of the results for this specific agent.

## Conclusions

In conclusion, our large retrospective cohort study showed that LMWH is not associated with a different risk of osteoporotic fracture than UFH among chronic hemodialysis patients when used for the extracorporeal circuit anticoagulation. Extracorporeal circuit anticoagulation during a hemodialysis session is mandatory, and ensuring that the increasing use of LMWH does not modify the risk of fractures is crucial.

## Supplementary information

**Additional file 1: Table S1.** Specific medical services billing codes for osteoporotic fractures by site. **Table S2.** Non-specific osteoporotic fracture billing codes and specific ICD-9/ICD-10 codes

## Data Availability

*The data that support the findings of this study are available from RAMQ, Med-Echo and from surveys conducted at participating centers but restrictions apply to the availability of these data, which were used under license for the current study, and so are not publicly available. Data cannot be shared due to a confidentiality agreement with the RAMQ and Ethics committees in each participating hospital.*

## Abbreviations

UFH: Unfractionated heparin
LMWH: Low-molecular-weight heparin
P-Y: Patient-year
CI: Confidence interval
HR: Hazard ratio
DOPPS: Dialysis Outcomes and Practice Patterns Study
OPG: Osteoprotegerin
RANK: Receptor activator of nuclear factor kB
RANKL: Receptor activator of nuclear factor kB ligand
RAMQ: Régie de l’assurance maladie du Québec
ICD: International Classification of Diseases
SD: Standard deviation
IQR: Interquartile range
USRDS: US Renal Data System
