# Supplementary material for: Osteoporotic fracture rates in chronic hemodialysis and effect of heparin exposure: a retrospective cohort study
Source: BMC Nephrol. 2020 Jul 9;21:261. doi: 10.1186/s12882-020-01916-4 (PMC7350680; doi:10.1186/s12882-020-01916-4)
Supplement: Supplementary file 1 — Additional file 1: Table S1. Specific medical services billing codes for osteoporotic fractures by site. Table S2. Non-specific osteoporotic fracture billing codes and specific ICD-9/ICD-10 codes [file 12882_2020_1916_MOESM1_ESM.docx]

Table S1: Specific medical services billing codes for osteoporotic fractures by site

| Fracture sites | Medical services billing codes |
| --- | --- |
| Spine | 9568, 9569, 9570, 9571, 9572, 9573, 9574, 9575, 9576, 9577, 2582, 2214 |
| Pelvis | 2578, 2581, 2584, 2580, 2579, 2583, 2771, 2772, 2773, 2707 |
| Shoulder fracture | 2537, 2559, 2534, 2536, 2370, 2407, 2833, 2824 |
| Humerus | 2608, 2633, 2911, 2609, 2640, 2634, 2610, 2912, 2635, 2921, 2605, 2568, 2630, 2606, 2598, 2631, 2655, 2607, 2632 |
| Elbow | 2404, 2039, 2834, 2222, 2230 |
| Forearm fracture | 2612, 2636, 2624, 2649, 2570, 2571, 2586, 2587, 2589, 2599, 2651, 2652, 2654, 2735, 2736, 2769, 2361, 2770 |
| Wrist | 2618, 2642, 2620, 2643, 2409, 2038, 2835 |
| Femur | 2695, 2638, 2715, 2687, 2716, 2714, 2739, 2740, 2688, 2689, 2742, 2667, 2690, 2673, 9589, 9590, 2410, 2333, 2849 |
| Knee | 9549, 2680, 2705, 2725, 2694, 2696, 9591, 9592, 2721, 2743 |
| Foot | 2710, 2744, 2730, 2734, 2709, 2729, 2711, 2691, 2731, 2732 |
| Ankle | 2708, 2727, 9542, 2886, 2887 |

Table S2: Non-specific osteoporotic fracture billing codes and specific ICD-9/ICD-10 codes

|  | Codes’ categories | Codes |
| --- | --- | --- |
| Orthopedic surgeon | Principal visit | 9150, 9162, 9127 |
|  | Consultation | 9160, 9170, 9165 |
|  | Follow-up | 9152, 9164, 9129 |
| Emergency physicians | Immobilization | 2800, 2863 |
|  | Examination | 15052, 15043, 15054, 15058, 15059, 15060, 15064, 15065, 15068, 15071 |
| General practitioner | Immobilization | 2800, 2863 |
|  | Examination | 8870, 0009, 9034, 9038, 0003, 9112, 0005, 8882, 0068, 9121, 8871, 0011, 9035, 9039, 0055, 9115, 0056, 9116, 0069, 9122, 8872, 0021, 9036, 9040, 0096, 9118, 0097, 9119, 0070, 9126, 0061, 9231, 0060, 9234, 0062, 9237 |
| ICD-9 Fracture codes | 8050, 8051, 8052, 8053, 8054, 8055, 8056, 8057, 8058, 8059, 8060, 8061, 8062, 8063, 8064, 8065, 8066, 8067, 8068, 8069, 8080, 8081, 8082, 8083, 8084, 8085, 8086, 8087, 8088, 8089, 8100, 8101, 8110, 8111, 8120, 8121, 8122, 8123, 8124, 8125, 8130, 8131, 8132, 8133, 8134, 8135, 8140, 8141, 8180, 8181, 8190, 8191, 8200, 8201, 8202, 8203, 8204, 8205, 8206, 8207, 8208, 8209, 8210, 8211, 8212, 8213, 8220, 8221, 8230, 8231, 8232, 8233, 8240, 8241, 8242, 8243, 8244, 8245, 8246, 8247, 8248, 8249, 8250, 8251, 8252, 8253, 8270, 8271, 8280, 8281, 8290, 8291, 9051, 9052, 9053, 9054, 9055 | |
| ICD-10 Fracture codes | S120.00, S121.00, S122.00, S122.10, S127.00, S129.00, S120.01, S121.01, S122.01, S122.11, S127.01, S129.01, S220.00, S220.10, S220.90, S221.00, S220.01, S220.11, S220.91, S221.01, S320.00, S320.10, S320.20, S320.30, S320.40, S320.90, S320.01, S320.11, S320.21, S320.31, S320.41, S320.91, S321.00, S322.00, S321.01, S322.01, T080, T081, S324.00, S324.01, S325.00, S325.01, S323.00, S327.00, S323.01, S327.01, S328.00, S328.01, S420.00, S420.20, S420.90, S420.01, S420.10, S420.11, S420.21, S420.91, S421.00, S421.10, S421.20, S421.80, S421.90, S421.01, S421.11, S421.21, S421.81, S421.91, S422.00, S422.10, S422.20, S422.80, S422.90, S428.00, S429.00, S422.01, S422.11, S422.21, S422.81, S422.91, S428.01, S429.01, S423.00, S423.90, S423.01, S423.91, S424.00, S424.10, S424.20, S424.30, S424.80, S424.90, S424.01, S424.11, S424.21, S424.31, S424.81, S424.91, S520.00, S520.10, S520.20, S520.80, S520.90, S521.00, S521.10, S521.20, S521.80, S521.90, S527.00, S528.00, S529.00, S520.01, S520.11, S520.21, S520.81, S520.91, S521.01, S521.11, S521.21, S521.81, S521.91, S527.01, S528.01, S529.01, S522.00, S523.00, S524.00, S522.01, S523.01, S524.01, S525.00, S525.80, S525.90, S526.00, S525.01, S525.81, S525.91, S526.01, S620.00, S621.00, S621.10, S621.20, S621.30, S621.40, S621.50, S621.60, S621.80, S621.90, S628.00, S620.01, S621.01, S621.11, S621.21, S621.31, S621.41, S621.51, S621.61, S621.81, S621.91, S628.01, S427.00, T022.0, T100, S427.01, T022.1, T101, T024.0, T024.1, S720.00, S720.10, S720.80, S720.90, S720.01, S720.11, S720.81, S720.91, S721.00, S721.90, S722.00, S721.01, S721.91, S722.01, S723.00, S727.00, S728.00, S729.00, S723.01, S727.01, S728.01, S729.01, S724.00, S724.10, S724.20, S724.90, S724.01, S724.11, S724.21, S724.91, S820.00, S820.01, S821.00, S824.00, S829.00, S821.01, S824.01, S829.01, S822.00, S822.01, S825.00, S828.20, S825.01, S828.21, S826.00, S826.01, S828.00, S828.01, S828.10, S828.11, S823.00, S828.90, S823.01, S828.91, S920.00, S920.01, S921.00, S922.00, S922.10, S922.20, S922.90, S923.00, S929.00 S921.01 S922.01, S922.11, S922.21, S922.91, S923.01, S929.01, S827.00, S927.00, T023.0, T120, S827.01, S927.01, T023.1, T121, T025.0, T026.0, T027.0, T028.0, T029.0, T025.1, T026.1, T027.1, T028.1, T029.1, T142.0, T142.1, T902, T911, T912, T921, T922, T931, T932, T940, T923, T933, T925, T935, T926, T936 | |
